# Loss of SYD-2 leads to decreased short-term memory in *Caenorhabditis elegans*

**DOI:** 10.17912/micropub.biology.002021

**Published:** 2026-01-27

**Authors:** Katherine Davies, Meclina Silva, Karla Vargas, Dayton Pierce

**Affiliations:** 1 Biology, Sacramento City College, Sacramento, CA, US

## Abstract

Learning and memory require tight regulation of communication between pre- and postsynaptic responses. SYD-2, the *C. elegans* ortholog of vertebrate liprin-α, is known for its presynaptic role in active zone maintenance but its role in learning and memory has not been previously investigated. Here, we present findings from 30-minute short-term aversive olfactory memory assay with *C. elegans* lacking SYD-2 ( *ok217* mutants). Wild-type (N2) worms exhibited robust learning and short-term memory for aversion to diacetyl. Despite its role in presynaptic maintenance, we found that the loss of SYD-2 had no effect on aversive learning but decreased short-term associative memory.

**Figure 1. syd-2 mutants exhibit normal learning but decreased short-term associative memory to diacetyl:**
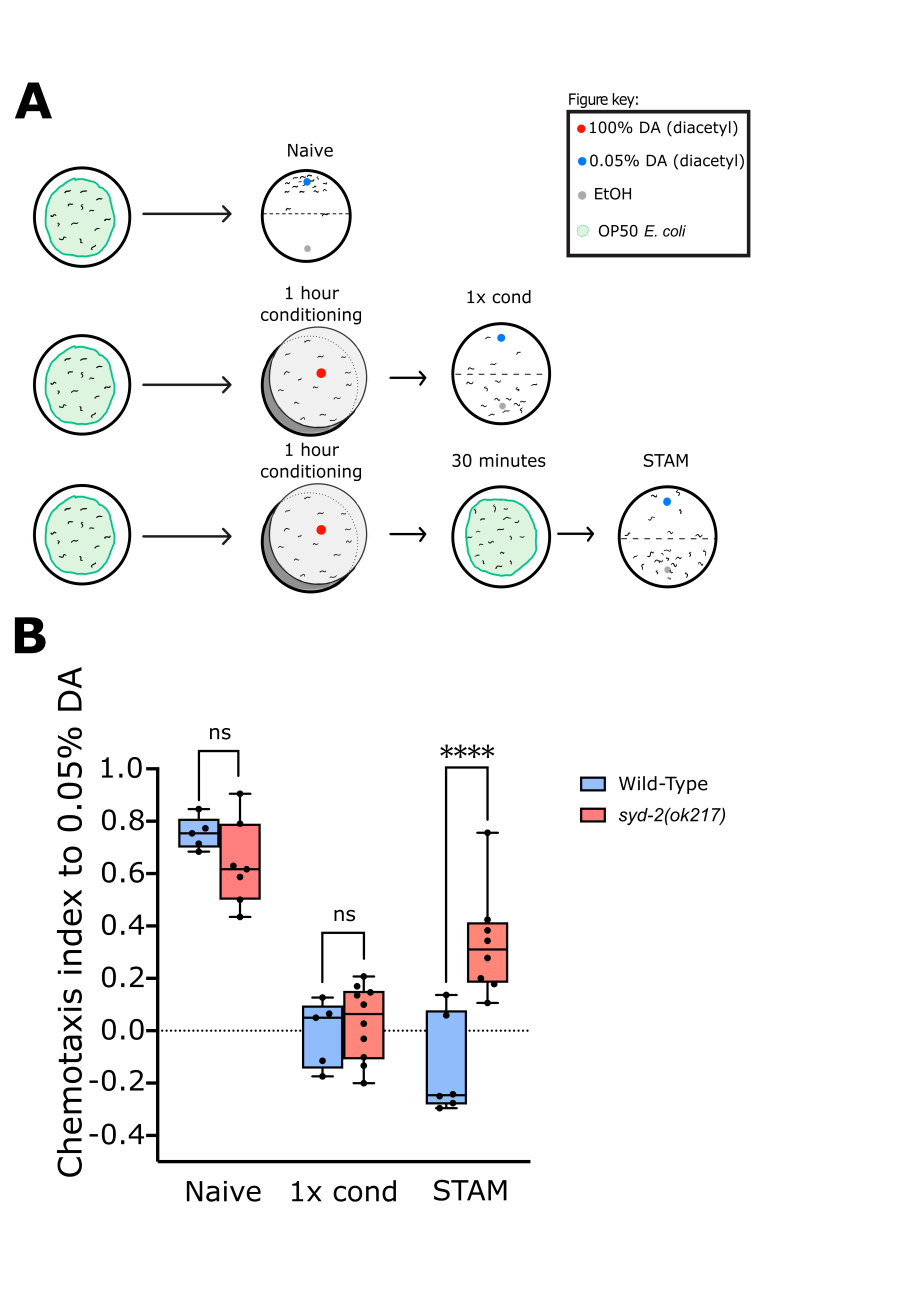
(A) Cartoon illustration of the associative chemotaxis learning and memory protocols for short-term associative memory (STAM). (B) Chemotaxis index quantification of day 1 adults of wild-type ( N2 ), and *syd-2 ( ok217 )* that were tested towards 0.05% diacetyl (DA) (naïve), worms that were conditioned for 1 hour with 100% DA without food (1x cond), or worms that were conditioned for 1 hour with 100% DA without food and allowed to recover for 30 minutes on a nematode growth medium (NGM) plate with OP50 food and then tested (STAM). The dot points indicate the average chemotaxis index of the plates for the experimental day (n = 5-10 trials). A two-way ANOVA with Bonferroni correction for multiple testing was used for statistical analysis. ****p < 0.0001. (Genotype: *F*_(1|35)_ = 6.684, Treatment: *F*_(2|35)_ = 67.26, Interaction: *F*_(2|35)_ = 12.16).

## Description

Learning and memory are closely tied to the strength of glutamatergic transmission which influences how neurons adapt and communicate (Riedel et al *.,* 2003; Peng et al *.,* 2011). Regulation of glutamatergic transmission strength requires synergistic changes at both synaptic compartments. At the postsynaptic compartment, processes that regulate synaptic glutamate receptor numbers like clustering, anchoring, and retention influence the postsynaptic response to glutamate. At the presynaptic compartment, the probability and amount of neurotransmitter release is regulated to adjust synaptic strength (Diering and Huganir 2018; Groc and Choquet 2020). When glutamate receptor localization at the postsynapse is disrupted, glutamatergic transmission is decreased ultimately leading to impaired learning and memory (Morrison and van der Kooy 2001; Hoerndli et al *.,* 2015; Hoerndli et al *.,* 2022). However, glutamatergic signaling also depends on the release of glutamate from the presynaptic active zone. During long-term potentiation (LTP), which is widely considered to be the molecular basis for learning and memory, there are structural changes that happen at both the pre- and postsynapse (Bender et al *.,* 2009; Bourne et al *.,* 2013; Bell et al *.,* 2014). Thus, an understanding of what regulates glutamate transmission at both the postsynaptic and presynaptic compartments is key to further understanding learning and memory.

During LTP, one structural change that occurs is that the presynaptic active zones increase in size and contain more presynaptic machinery to increase the amount of neurotransmitter released (Bell et al *.,* 2014; Harris 2025). The molecular motor KIF1A has a key role in regulating synaptic neurotransmitter release from the active zone. (Zhang et al *.,* 2017; Vitet et al *.,* 2023 **)** . The *Caenorhabditis elegans* ( *C. elegans* ) homologue of KIF1A, UNC-104 , has been shown to be important in presynaptic vesicle transport (Nadiminti et al *.,* 2024). Interestingly, UNC-104 's function is influenced by adapter molecules including the scaffolding protein SYD-2 (Nadiminti et al *.,* 2024). SYD-2 is well-established for its crucial role in presynaptic organization, specifically in regulating active zone size and vesicle clustering (Zhen and Jin 1999; Dai et al *.,* 2006). Despite this, SYD-2 's role in learning and memory has not been explored directly, therefore we sought out to answer the question: is SYD-2 required for short-term associative memory in *C. elegans* ?

To study this question, we tested short-term aversive olfactory memory in *syd-2* null worms ( *ok217* ) (Wagner et. al *.,* 2009). Worms were trained to associate the odorant diacetyl (a natural attractant) with starvation, and we tested odorant preference/aversion in both naïve and trained worms using a chemotaxis assay. This experiment tests whether worms can associate the smell of DA with the averse effect of starvation (no food). Traditionally, diacetyl-based short-term associative memory (STAM) experiments have been performed where following conditioning, worms are transferred to a fresh NGM + OP50 for a 1-hour period to allow for memory consolidation. However, short-term memory in *C. elegans* has a time-dependent aspect that is affected by presynaptic genes such as *acy-1* which modulates neurotransmitter release in *C. elegans* and is crucial in regulating memory just 30 minutes post training (Schade et al *.,* 2005; Stein and Murphy, 2014; Cohen and Rabinowitch 2024; Zhu et al *.,* 2025). If presynaptic genes can affect memory 30 minutes after training and SYD-2 is known to impact the presynaptic site, it's possible that SYD-2 is required for memory consolidation in this time frame. To test for 30-minute STAM, 1-day old worms were trained then transferred to a fresh NGM plate with OP50 for 30 minutes to allow for memory consolidation. After this time, we tested DA chemotaxis and found that *syd-2* mutants had no observable defect in movement or naïve chemoattraction towards the DA compared to N2 wild-type controls (“Naïve”, Figure 1B ). When testing for learning of the negative association of DA to starvation, loss of SYD-2 did not show a difference between wild-type chemotaxis indexes, indicating SYD-2 is not required for aversive learning to DA (“1x cond”, Figure 1B ). Interestingly, when testing for 30-minute short-term memory, the chemotaxis index was increased in *syd-2* null mutants compared to wild-type suggesting a reduction in short-term memory and that SYD-2 is required for 30-minute short-term memory (“STAM”, Figure 1B ).

The known network of neurons important in olfactory associative memory are RIA, AVE, AVD, and AVA (Stetak et al *.,* 2009; Rahmani and Chew 2021). RIA interneurons receive input from sensory neurons which then can relay signals to AVE, AVD, and AVA. Although RIA does not directly synapse onto AVE, AVD, or AVA, it works upstream of these neurons to facilitate olfactory associative learning (Stetak et al *.,* 2009). RIA is a glutamatergic interneuron whose direct postsynaptic connections are not well defined in learning and memory (WormAtlas). Interestingly, SYD-2 shows a strong expression pattern in RIA in adult worms (Taylor et al *.,* 2021). This could suggest that there is impaired glutamatergic signaling at the presynapse in RIA. Surprisingly, when RIA is completely ablated, worms do not learn starvation-induced aversion of DA (Stetak et al *.,* 2009). The intact learning seen in *syd-2* mutants may suggest that SYD-2 is not required for short-term plasticity mechanisms (“1x cond”, Figure 1B ) but may be involved in regulating longer-term (post consolidation) changes in glutamate release.

Because we did not observe measurable changes in learning in *syd-2* mutants and it has been shown that SYD-2 is highly expressed in the postsynaptic glutamatergic neuron AVA, it is also possible that SYD-2 is involved postsynaptically such as in controlling glutamate receptor localization to synapses. It is becoming more evident that pathways affecting the trafficking of AMPA subtype of glutamate receptors (AMPARs) to postsynaptic sites of AVA are critical to learning and memory in *C. elegans* (Rahmani and Chew 2021; Stetak et al *.,* 2024). The vertebrate homologue of SYD-2 , liprin-α, is known to associate to the kinesin-3 motor KIF1A while *Drosophila* liprin-α has shown to have robust association with kinesin-1 heavy chain (KHC) (Miller et al *.,* 2005). The sole kinesin-1 heavy chain in *C. elegans* ( UNC-116 ) is crucial for AMPAR transport to synapses in AVA (Hoerndli et al *.,* 2013, Hoerndli et al *.,* 2015). Sequence homology between *Drosophila* KHC and *C. elegans* UNC-116 reveals ~60% conservation and thus it is possible that SYD-2 interacts with UNC-116 in *C. elegans* to affect AMPAR numbers in AVA. In summary, it is also possible that SYD-2 is involved in regulating postsynaptic strength via its role in glutamate receptor transport which may additionally explain our observation of short-term memory impairment in *syd-2* mutants.

## Methods

**Strains and Strain Maintenance**

Worms were maintained on standard NGM agar plates seeded with OP50 *E. coli* at 20°C (Brenner 1974).

**Table:** 

**Strain**	**Genotype**
N2	Wild-Type
ZM607	*syd-2* ( *ok217* )

**Chemotaxis Assay**

Chemotaxis assays for the naïve protocol were performed on 10 cm assay plates as described (Cesar and Morud 2025). Briefly, L4 worms were picked to seeded NGM plates seeded with OP50 on day 1 and chemotaxis plates (CTX) were prepared and left to dry in a laminar flow hood. On day 4, between 50-100 L4 worms were transferred to fresh NGM with OP50 . On day 5, worms were washed with 2mL of M9 buffer into 1.5mL microcentrifuge tubes using a glass pipette. Worms were allowed to pellet by gravity for 5 minutes and then supernatant was removed and washed with 1mL of fresh M9 buffer. This process was repeated 3 more times to ensure no leftover OP50 bacteria was in the mixture. During the second to last wash, CTX plates were labeled with a dotted line in the center of a plate and two solid lines 0.5cm from the center line on each side. Two marked circles were drawn on either side of the plate, each 1 cm in diameter and 0.5 cm from the end of the plate.&nbsp; Concentrated diacetyl was diluted 1:2000 in 96% ethanol and mixed in a 1:1 ratio of 1M sodium azide and used as the “test” spot. 96% ethanol mixed with 1:1 sodium azide was used as the “control” spot. 2mL of the “test” was pipetted at the test spot and 2mL of the “control” was pipetted at the control spot. After the washes, the supernatant was removed and the worm pellet was transferred to the center of the CTX testing plates with a glass pipette. Kimtech wipes were used to carefully wick excess liquid to allow worms to crawl. The plates were wrapped in parafilm and kept in the flow hood for 1.5 hours and worms were counted after this time period. Chemotaxis index was then calculated as: Chemotaxis index = (Number of worms at “test” − Number of worms at “control”)/(Total number of worms on the plate) as previously reported (Bargmann et al *.,* 1993).

A conditioning protocol modified from (Stetak et al *.,* 2009) was used. The same 5-day procedure as mentioned above was the same except on Day 5, worms were washed with M9 buffer and transferred to a “training plate” (a CTX plate devoid of OP50 or sodium azide) that had 2mL of 100% diacetyl spotted on parafilm attached to the inside of the lid. After one hour on this “training plate”, worms were washed with M9 buffer and transferred to a marked experimental plate with the “test” and “control” odorants for 1.5 hours. Then, worms were quickly counted manually and chemotaxis index was calculated.

For testing STAM, worms were conditioned for 1 hour with 100% diacetyl as described above. Worms were then washed with M9 buffer and transferred to a fresh NGM with OP50 plate for 30 minutes. These worms were collected with M9 buffer in 1.5mL microcentrifuge tubes and washed with M9 buffer 3 times to remove excess OP50 . Worms were transferred to the center of an experimental test plate with the “test” and “control” odorants for 1.5 hours. Worms were counted and chemotaxis index was calculated.

All experimental plates were counted blind to genotype and only plates containing 40+ worms were counted and considered acceptable results.

**Data Analysis:** All assays were conducted with 2-4 plates for each strain per day per experimental protocol and the average chemotaxis assay per day was plotted replicated 5-10 trials per protocol. Statistical analysis of our datasets was determined by using a two-way ANOVA with Bonferroni correction for multiple testing. Conditions tested: genotype, treatment, interaction in GraphPad Prism 10. All graphs were produced by GraphPad Prism 10 and exported to the program Inkscape for final processing.

## Reagents

**Materials:** Standard OP50 *E. coli;* NGM plates; Chemotaxis plates (5 mM KH2PO4/K2HPO4 [pH 6.0], 1 mM CaCl2, 1 mM MgSO4, 2% agar); Diacetyl (Sigma-Aldrich #B85307-5ML); 96% ethanol; Sodium azide (ThermoFisher J21610.22); M9 buffer.
